# Systematic development of a communication skills training course for physicians performing work disability assessments: from evidence to practice

**DOI:** 10.1186/1472-6920-11-28

**Published:** 2011-06-03

**Authors:** H Jolanda van Rijssen, Antonius JM Schellart, Johannes R Anema, Wout EL de Boer, Allard J van der Beek

**Affiliations:** 1 Department of Public and Occupational Health, EMGO Institute for Health and Care Research, VU University Medical Center, Amsterdam, The Netherlands; 2 At the time: TNO Quality of Life, Hoofddorp, The Netherlands; 3 Research Center for Insurance Medicine, collaboration between AMC-UMCG-UWV-VUmc, Amsterdam, The Netherlands; 4 Academy of Swiss Insurance Medicine, University Hospital, Basel, Switzerland

## Abstract

**Background:**

Physicians require specific communication skills, because the face-to-face contact with their patients is an important source of information. Although physicians who perform work disability assessments attend some communication-related training courses during their professional education, no specialised and evidence-based communication skills training course is available for them. Therefore, the objectives of this study were: 1) to systematically develop a training course aimed at improving the communication skills of physicians during work disability assessment interviews with disability claimants, and 2) to plan an evaluation of the training course.

**Methods:**

A physician-tailored communication skills training course was developed, according to the six steps of the Intervention Mapping protocol. Data were collected from questionnaire studies among physicians and claimants, a focus group study among physicians, a systematic review of the literature, and meetings with various experts. Determinants and performance objectives were formulated. A concept version of the training course was discussed with several experts before the final training course programme was established. The evaluation plan was developed by consulting experts, social insurance physicians, researchers, and policy-makers, and discussing with them the options for evaluation.

**Results:**

A two-day post-graduate communication skills training course was developed, aimed at improving professional communication during work disability assessment interviews. Special focus was on active teaching strategies, such as practising the skills in role-play. An adoption and implementation plan was formulated, in which the infrastructure of the educational department of the institute that employs the physicians was utilised. Improvement in the skills and knowledge of the physicians who will participate in the training course will be evaluated in a randomised controlled trial.

**Conclusions:**

The feasibility and practical relevance of the communication skills training course that was developed seem promising. Such a course may be relevant for physicians in many countries who perform work disability assessments. The development of the first training course of this type represents an important advancement in this field.

## Background

Physicians require specific communication skills, because the face-to-face contact with their patients is an important source of information. Likewise, for physicians who perform work disability assessments, the interview with the claimant is in many countries an important source of information [1,2]. This interview gives the claimant the opportunity to clarify and substantiate his or her claim, and it also gives the physician the opportunity to observe the claimant's behaviour, to discuss the claimant's disabilities and consequences thereof, and to reassure the claimant when necessary. Both the content and the process of the interview are important. The content is important because the physician's goal is to obtain all the necessary information for the disability assessment and to make the right decision. The process is important because the claimant should feel that he or she is being taken seriously and treated fairly, and should be willing to provide information and accept the outcome [3-5]. In this paper we focus on the process of the interview, and especially the communication between the physician and the claimant. Communication is defined as face-to-face contact between physician and claimant, aimed at verbal and non-verbal two-directional exchange of information (including facts, opinions, and feelings, both conscious and unconscious). Although communication behaviour, such as checking understanding and summarising information, is linked to patient trust and satisfaction, and is important for many aspects of clinical care, research has shown that physicians rarely check whether patients comprehend the information [6]. Research has also shown that the degree of effectiveness of the physician-patient communication determines the accuracy and completeness of the information that the physician receives from the patient [7,8]. Furthermore, good communication increases the likelihood that patients accept and follow the advice of physicians [9].

Numerous programmes for training physicians in communication skills exist [10], some of which are intended for physicians in specific fields of care, such as cancer care [11-13]. No evidence-based training course was found for physicians performing work disability assessments. The assessment interviews differ from interviews held by other physicians in that they are not primarily aimed at cure or care for patients, but at assessing the work capacities and incapacities of disability claimants and scrutinising their reasons for not working. Because the physician and the claimant do not necessarily have a joint aim during the assessment interview, physicians have to deal with several ethical issues. These issues may, for example, be related to the fact that the assessment is likely to have both material (e.g. financial) and immaterial (e.g. emotional) consequences for the claimant, there is a lot at stake for the claimant, and the claimants is more or less obligated to attend the assessment. Moreover, the time that is available to gather all the necessary information for this assessment is generally short. Because of these specific aspects of the assessment interviews, good communication is essential [14]. The specific demands for physicians and the central role of communication in these assessments, call for a specialised communication skills training course. Physicians do receive communication training during their professional education, but to date there is no evidence-based, post-graduate training course that is tailored to work disability assessments. Therefore, this paper describes the systematic development of a communication skills training course aimed at improving the communication behaviour of physicians during disability assessment interviews.

## Methods

To ensure that the training course would be tailored to its users, and would addresses the communication needs of all directly concerned stakeholders, it was developed according to the Intervention Mapping (IM) protocol [15]. This protocol is generally used for the development of health promotion plans, but it can also be applied in the development of other interventions [16,17]. The IM protocol has three main information inputs: literature searches, theories, and newly collected data. The protocol consists of six steps: (1) assessing needs, (2) formulating programme objectives, (3) selecting theory-based methods and practical strategies, (4) designing the programme plan, (5) designing the adoption and implementation plan, and (6) designing the evaluation plan. We have described the methods for each of these six steps below. In several steps we used the results of our prior research in this area, indicated by referring to accompanying scientific publications. According to the Dutch law, no ethical approval was needed for this study and the prior research that is referred to in this study. The participants received no intervention and were asked no medical information. For the (prior) questionnaire studies, all claimants and physicians gave informed consent.

### Step 1: Needs assessment

The first step in IM was to identify the needs of stakeholders for a communication skills training course for physicians who perform work disability assessments. According to the extended script model [1], there are three main stakeholders within the Dutch system (this study took place in the Netherlands) that should be consulted. The first stakeholder was the Dutch Institute of Employee Benefit Schemes, which is the institute that employs most of the physicians performing work disability assessments for entitlement to benefits (further referred to as 'the Institute'). The second group of stakeholders consisted of medical disability claimants (in this study, employees who had been sick-listed for almost two years, applying for a long-term work disability benefit). The third group of stakeholders consisted of physicians who were specialised in performing work disability assessments (in this study, social insurance physicians). Although the practice varies considerably among countries, long-term work disability assessments are usually performed by specialised social insurance physicians [1,4].

Firstly, we identified the needs of the Institute by consulting the four policy-makers with the most expertise of physician-claimant communication, and studying reports and publications of the Institute and allied organizations. Secondly, we assessed the needs of the claimants (n = 56) in a survey, by means of an open-ended question, asking for comments on the communication during an assessment interview they had recently attended [18]. Thirdly, we used the results from a focus group study among social insurance physicians (n = 22) to assess the needs of the physicians [19]. By combining the needs of these three stakeholders, the desired programme outcome for the communication skills training course was determined.

### Step 2: Programme objectives

Having assessed the needs in the first step, the second step in IM is to formulate the aim of the programme, the programme objectives, the performance objectives, and the change objectives. The aim was deduced from the combined needs of the stakeholders. In a brainstorming session, the programme objectives were formulated, based on the aim, with additional input from result matrices. Input for these matrices were the combined results from two questionnaire studies among social insurance physicians [20], two questionnaire studies among work disability claimants [18], and a focus group study among social insurance physicians (n = 22) [19]. The first questionnaire study among social insurance physicians (n = 146) assessed their general preferences in the communication during work disability assessment interviews and the psychosocial determinants of their communication behaviour [20]. The second questionnaire study among social insurance physicians (n = 56) assessed their opinion about and satisfaction with the communication during 10 assessment interviews. The first questionnaire study among claimants (n = 63) assessed their general preferences in the communication during work disability assessment interviews and physician-patient encounters in general, and the psychosocial determinants of their communication behaviour. The second questionnaire study among claimants (n = 56) assessed their opinion about and satisfaction with the communication during a recently attended interview. In addition to the results for each questionnaire separately, analyses of the combined results of both second questionnaires were performed to obtain insight into agreements and differences of opinion about the communication between physicians and claimants [21]. Additional analyses were also performed, in which the data from all four questionnaires were combined (n = 28 physicians, n = 53 claimants), to assess at positive agreement between physicians and claimants with regard to the communication during the interview in more detail.

We formulated performance objectives describing the type of behaviour the physicians should be able to adopt after they had participated in the training course, by translating the programme objectives into more specific training goals, based on matrices. This was done in brainstorming sessions attended by all authors and two experts on the communication skills of social insurance physicians in the Educational Department of the Institute. These experts agreed to collaborate more intensely with the authors in the development and implementation of the training course, in order to ensure practical relevance and feasibility of the aims and objectives. The change objectives at organisational level were derived from the performance objectives and formulated by the authors, after consulting the social insurance physicians in the focus group study and two policy-makers at the Institute.

### Step 3: Selecting theory-based methods and practical strategies

Having formulated the performance objectives with each determinant and the change objectives in the second step, the third step in IM is to identify theory-based methods and practical strategies that could effect changes in the determinants of the communication behaviour of social insurance physicians.

The methods and strategies were identified on the basis of findings of a previous systematic review of the most effective strategies for teaching communication skills to physicians [10]. We also took the theoretical framework underlying our questionnaire studies [5] as a starting point to search the literature for an appropriate cognitive or behavioural model for teaching communication skills. To select appropriate methods and strategies from the systematic review and the theoretical models, we organised brainstorming sessions attended by some of the authors and the two experts in communication skills of social insurance physicians at the Institute. Remarks made by the physicians in the afore-mentioned focus group studies, were also compared to the findings. We then made a matrix to determine the most appropriate methods, strategies, and relevant materials for each behavioural determinant.

### Step 4: Programme plan

After formulating theoretical methods and practical strategies in the third step, the fourth step in IM is to evaluate the established content of the programme in relationship to the context of the programme and the intended participants. From the tables and matrices formulated in the previous steps, those performance objectives that could be addressed in a short training course were selected by the authors and the experts. A concept training course was developed, and subsequently discussed and evaluated to assess its strengths and weaknesses. This evaluation was first made with three additional experts in the development and/or provision of training programmes for social insurance physicians, and successively in a group of 15 social insurance physicians. The authors presented the research results and concept versions of the training course in meetings, and asked the physicians to comment on its content and to provide further suggestions for improvement. We then discussed the comments and suggestions from the meetings with the two experts, to establish the final training programme.

### Step 5: Adoption and implementation plan

After the programme plan was established in step 4, the fifth step in IM was to develop a plan for the adoption and implementation of the training course in practice. This was done in collaboration with the two experts from the Institute's Educational Department. A plan was made to promote the training course to potential participants, both top-down with the assistance of the managerial staff, and bottom-up by directly approaching physicians. This plan concerned who to approach, at which moment in time, and in which way (e.g. by means of a presentation, e-mail, both directly and indirectly), also including people and institutions that might be able to facilitate in the adoption, for example by raising enthusiasm among physicians. The aim of the plan was to reach all physicians with experience in work disability assessments and working for the Institute. The implementation was supported by a manual which was developed for the teachers of the training course.

### Step 6: Evaluation plan

When the plans for adoption and implementation had been completed, the sixth step in IM was to formulate an evaluation plan, taking all findings from the prior steps into account. During the development of the training course, we realised that it was impossible to implement our original idea to evaluate the training course on a large scale in practice in a randomised controlled trial (RCT), with as primary outcomes the claimants' acceptance of the physician's conclusions and satisfaction with the communication. This was mainly due to organisational changes within the Institute, which limited the number of eligible participants and resulted in huge practical problems. To formulate a new evaluation plan, we therefore organised a brainstorming session with all the authors to generate alternative evaluation plans, including other RCT designs and alternative designs. Subsequently, all these plans were presented - with their advantages and disadvantages - to 30 researchers and to 15 social insurance physicians and researchers. They commented on the plans and explained what their choice would be. After consulting the staff and policy makers of the Institute with regard to feasibility issues, the authors made the final decision on the evaluation plan. The Medical Ethics Committee of the VU University Medical Center informed us that this evaluation study would not need ethical approval.

Having formulated the evaluation plan, the required measurement instruments had to be developed, taking into account that our study would only be financially feasible if all measurements were obtained with questionnaires. The literature was searched for available questionnaires, and an expert on measurement instruments for communication skills was consulted. The resulting questionnaires were pilot-tested by four social insurance physicians to assess comprehensibility and relevance, and by two researchers who were familiar with the intervention to assess whether the contents of the training course and the questionnaires matched. We made the final choice of questionnaires, taking their remarks into consideration, as well as the time needed to complete the questionnaires.

## Results

### What physicians need to learn regarding communication

The Institute states on its website that it strives to "excel as a provider of social services by focussing attention on the claimant" (http://www.uwv.nl, accessed 18 June 2010). This includes showing interest in claimants and respecting them, being clear about promises and expectations, and delivering the appropriate services. Other internal publications confirm that delivering good insurance-medical care, which includes correct physician-claimant communication, is the main aim of the Institute. The Institute also considers claimant satisfaction important, and tries to minimise the number of complaints, objections, and appeals that claimants file. Therefore, policy-makers at the Institute would favour a training course aimed at increasing the services for claimants by improving the professional communication behaviour of physicians during disability assessment interviews.

From the claimant's perspective, difficulties in communication during already stressful interviews may have a considerable impact. According to their responses to our questions, the claimants were of the opinion that in a communication skills training course it is especially important that physicians: (1) provide clear and complete information about the assessment, the interview, and the findings, (2) show empathy, for example, with regard to the tension that the assessment may cause in the claimant, (3) take the claimant seriously, by limiting the influence of preconceived notions and suggestive questions, and (4) take the necessary time and make the necessary preparations, in order to obtain sufficient prior knowledge about the disabilities of the claimant.

In the focus group meetings the physicians themselves indicated that the time that is available per claimant is limited, and therefore they would like to learn how to perform the interview more efficiently, while maintaining a professional method of communication. The fact that the physicians reported that they experienced very few communication problems, while claimants had many, might indicate a lack of awareness on the part of the physician. With regard to this, the physicians indicated that they wished to minimise the influence on their communication behaviour of their unconscious feelings and opinions with regard to the claimants (e.g. be aware of counter-transference, recognising the effect of claimant behaviour on their own behaviour).

### Defining professional communication

In brainstorming sessions, combining the results of the needs assessment, and using matrices to summarise and structure all findings, we formulated the main aim of the communication skills training course: social insurance physicians should communicate in a professional way, as a consequence of which both claimants and physicians experience less difficulty in the communication. The communication behaviour of physicians was considered to be 'professional' if it is in accordance with the three programme objectives. These programme objectives and the main research findings that resulted in these objectives, are summarised in Table 1.

**Table 1:** Summary of the main research findings from the matrices for the translation into programme objectives

	Main research findings	Programme objectives
1	- Social insurance physicians (SIPs) have little awareness of the effects of claimant (CL) behaviour on their own communication behaviour, and vice versa. - SIPs are often unable to accurately assess CLs' opinions about the communication. - SIPs assume that CLs' opinions are more positive than those opinions actually are. - Barriers that SIPs may experience in interaction with CLs, may influence the communication. - When the behaviour of SIPs is too self-assured, this may hinder the communication.	Physicians are aware of the influences of their own feelings and assumptions about claimants on their behaviour when communicating with those claimants, and they minimise negative influences.

2	- SIPs should communicate clearly. - SIPs should respond empathically to CLs (affective, emotion-oriented communication), in addition to focussing on the content (instrumental, task-oriented communication). - The former applies especially to CLs who SIPs assume to have little functional capacity. - CLs have a more positive opinion about the communication when the physician pays more attention to them (e.g. is transparent, provides clear explanations, discusses their work and personal situation). - The introduction of the interview is important, because it provides the basis for the rest of the interview. - In interviews with CLs with a lower level of education, with little self-reported communication skills, and with little social support from family, friends, and acquaintances, SIPs need to pay special attention to the exchange of information and their listening behaviour.	Physicians communicate efficiently, clearly, and empathically, attuned to claimants.

3	- When SIPs are transparent and clear, providing information about findings and conclusions, this may prevent unpleasant reactions from CLs that SIPs fear. - Although most SIPs reported that they explained their conclusions to the CLs, many CLs reported that this did not happen. - CLs often find the SIP's conclusion unclear or difficult to understand.	Physicians meet claimants' needs for information and empathy in their communication behaviour when they discuss their findings without compromising the assessment.

### Support from the Institute

We found that change objectives should concern obtaining support from the Institute, to make it possible to implement the training course. Support includes practical support (e.g. financing, location of the training course, offering physicians the time to attend), as well as 'emotional' support (e.g. making it known that the Institute finds the training course and its subject important, motivating physicians to join). Acquiring accreditation of continuing medical education for this new training course was an important change objective to give the training course an official status within the Institute and its Educational Department. This also made it possible for the training course to be embedded in the Educational Department, including the use of all the available facilities.

### Identifying training goals

Next, in brainstorming sessions, using the matrices and other research findings, we specified six performance objectives with regard to two determinants: (a) awareness and knowledge about the communication behaviour, and (b) and communication skills. We decided on these determinants because several reviews have included them as important determinants [12,22,23]. Table 2 provides a summary of the performance objectives.

**Table 2:** Programme objectives (1-3) related to performance objectives in social insurance physicians' knowledge, awareness, and skills

Programme	Performance objectives for SIPs	
	
objectives	a. Knowledge/awareness	b. Skills
**1. SIP is aware of the influence of own feelings and assumptions, minimising negative influences**	Social insurance physician (SIP) knows that there is a constant interaction between SIP and claimant (CL) communication behaviour, with regard to both content and process.	SIP switches between content and process in the communication, geared to CL's verbal and non-verbal behaviour (e.g. reflects on CL's feelings, labels non-verbal behaviour).
	
	SIP is aware of the influence of own communication preferences and own state of mind in relation to CL's verbal and non-verbal behaviour.	SIP signals the effect of own disturbing feelings and assumptions in relation to CL behaviour, and takes this into account.
	
	SIP knows the general rules of giving adequate feedback.	SIP gives appropriate feedback about CL's behaviour, especially if it disturbs SIP.

**2. SIP communicates efficiently, clearly, and empathically, attuned to claimant**	SIP knows what instrumental (task-oriented, content-focussed) and empathic (affective, process-oriented) behaviour is, what the differences are, and when to use which.	SIP switches between instrumental and empathic behaviour during the interview.
	
	SIP knows the essential elements of a first-time introduction, including an explanation of the aim of an assessment interview.	SIP uses the essential elements of a first-time introduction, including an clear explanation of the aim of the assessment interview.
	
	SIP knows which general communication skills exist (e.g. asking open-ended/closed questions, listening, summarising, providing regular breathing spaces), and when to use which.	SIP uses general communication skills, each at the appropriate moments resulting in clarity in the communication.

**3. SIP meets CL's needs for information and empathy when discussing the findings**	SIP knows the importance of actually mentioning the conclusions to the CL.	SIP mentions and explains the conclusions clearly to the CL.
	
	SIP knows the essential elements of sharing and explaining a conclusion (i.e. the elements of a bad news conversation) [31,32].	SIP applies all essential elements (e.g. giving CL an opportunity to respond) when sharing and explaining conclusions to CL.
	
	SIP knows how to apply the knowledge of objectives 1 and 2 (listed above) when explaining conclusions to CL.	SIP applies the skills of objectives 1 and 2 (listed above) when explaining conclusions to CL.

### Choosing active teaching strategies

The systematic review showed that active strategies, including a lot of practice of the skills, for example in role-play with structured feedback, is a good method for teaching physicians communication skills. Moreover, interactive discussion in small groups, focusing on claimant communication, should be preferred over lectures. The additional literature search for a behavioural model showed that the findings of the systematic review were in line with Bandura's Social-Cognitive Theory (SCT) [24,25] and Kolb's model of learning styles [26]. According to the SCT, learning is facilitated by observing others (observational learning) and imitating examples of behaviour (modelling). Moreover, it considers performing behaviour and receiving feedback to be important strategies for acquiring behavioural skills. The Kolb model of learning styles consists of a processing continuum from active learning ('doing') to reflective observation ('watching'), combined with a perception continuum from abstract conceptualisation ('thinking') to concrete experience ('feeling'). Both models were even more useful because the experts considered both practice and experience to be important. The importance of 'doing' from the model of learning styles was emphasised by the review findings and the opinions of the physicians in the focus group meetings, who stated that communication is often easier in theory than it is in practice. These physicians also indicated a need for 'watching': getting theoretical examples (e.g. theory on communication techniques or how to communicate conclusions) and practical communication examples (e.g. from role models or peers). The need for theory stressed the need for 'thinking', as did the finding that physicians would appreciate a structured list of short and clear hints in the training course. Although 'feeling' was not mentioned by the physicians as a necessary ingredient, the experts we consulted agreed that this ingredient was needed for the learning process during the training course. The theoretical methods, practical strategies, and materials needed to increase knowledge and skills are summarised in Table 3 for each of the determinants and programme objectives.

**Table 3:** Theoretical methods, practical strategies, and tools/materials needed to change the behavioural determinants of physicians' communication

Determinant & programme objective	Theory-based method	Practical strategy	Tools/materials
**General, all determinants**	- Abstract conceptualisation (*thinking*) - Reflective observation (*watching*)	Providing written information	Hand-outs on all subjects

**Knowledge/awareness of influences of own feelings**	Abstract conceptualisation (*thinking*)	Providing verbal and written information	- Theoretical model of interpersonal communication - Theoretical model of giving feedback

**Skills in minimising negative influences**	- Concrete experience (*feeling*) - Active experimentation (*doing*)	- Guided practice with feedback - Providing examples (peer modelling)	- Group practice ('playground') - Feedback from teachers and peers

**Knowledge/awareness of communication attuned to CLs**	- Abstract conceptualisation (*thinking*)	- Guided group brainstorming - Providing verbal and written information	- Group discussion - Checklist on flip-over - Clarification by teachers

**Skills in communication attuned to CLs**	- Active experimentation (*doing*) - Reflective observation (*watching*)	- Guided practice with feedback - Providing examples (peer modelling)	- Group practice ('playground') - Feedback from teachers and peers

**Knowledge/awareness of meeting CL's needs when discussing findings**	- Abstract conceptualisation (*thinking*) - Reflective observation (*watching*)	- Providing verbal information - Discussing verbal information	- Theoretical model of discussing conclusions - Group discussion

**Skills in meeting CL's needs when discussing findings**	- Active experimentation (*doing*) - Reflective observation (*watching*)	- Guided practice with feedback - Providing examples (peer modelling)	- Group practice ('playground') - Feedback from teachers and peers

### Main approach for each addressed topic

It was agreed that these methods and strategies would be incorporated in the training course in the following way. In order to create a clear and safe situation for the participants, the teachers introduced each subject that was addressed in the training course by providing the relevant theoretical information, when available, in a theoretical model. Guided practice with feedback was the main ingredient of the training course. A practical situation was chosen for role-play, with one of the physicians playing the role of the physician and a teacher playing the role of the claimant. All the other participants observed, and afterwards the role-playing physician reflected on the performance, followed by feedback and suggestions from the teachers and the other physicians. The physician can be allowed to continue, or to try again, or another physician can be asked to give it a try. This group practice was referred to as 'the playground', in order to stress the opportunity it offers to practice in a safe environment, instead of creating a scary situation in which the participants judge each other's skills. To facilitate the learning process, checklists (e.g. with the essential elements of an introduction) were made, together with the participants, on large sheets of paper, the teachers making sure that all relevant items were included. To promote knowledge, the teachers integrated a top 10 of the most important research findings from the questionnaire studies and the focus group study (see step 2) in the training course. We included a binder with handouts, to remind physicians what they had learned and to enable them to look up information afterward the course.

### The order in which the topics are addressed

As advised by the three experts and the 15 social insurance physicians, the performance objectives were addressed in the training course in the same order as in a disability assessment interview. Following the sequence of the three programme objectives, the medical disability assessment was divided into three stages for this purpose: (1) preparation and introduction, (2) gathering information for the assessment, and (3) discussion about the conclusions and closing the interview. During this fourth IM step, no significant changes needed to be made in the previously established content of the training course. A summary of the training programme is presented in Additional file 1: Summary of the training programme. The training course programme was sent to the Dutch Social Physicians Registration Committee for official approval and accreditation for continuing medical education.

### Practical issues

From the evaluation of the concept training course in the discussion sessions, it was decided that the training course would be called 'Professional Claimant Communication', because during the development phase communicating with claimants in a professional way became the central theme of the course. In order to create a positive and safe learning environment, it was agreed that the main explicit focus of the training course should be on *professional *style and not on reducing the complaints of claimants.

According to the experts, a two-days period was sufficient to teach the physicians the basic knowledge, awareness, and communication skills. Moreover, in large-scale implementation it would probably be difficult for physicians to spend more than two successive days at a training course.

Physicians do realise (from practice and education) that they perform the work disability assessments primarily within the framework of the law and regulations. Therefore, they have both a societal task (the disability benefit is a form of social insurance) and a task in their approach of the claimant. Because of this combination, ethical dilemmas or issues can arise. For example, physicians should realise that it is generally no free choice of claimants to attend an assessment interview (people have the right to claim for a disability benefit, but once claiming a benefit they have several duties, such as giving information about their health, medical disabilities, and capacities). In the training course we have developed, ethical issues will become visible and will be discussed during the role-play and group discussions. These will then be addressed in the 'standard' way: sharing experiences, practicing different approaches towards the claimant, and experimenting with new approaches. Because of the importance of ethical dilemmas for social insurance physicians, we made sure that the teachers of the course had enough expertise with regard to ethics.

The active and interactive design of the training course limited the number of participants per group to 12 (which is a common number in such training courses). It was established that the inclusion criteria for participation in the training course were that the physicians worked as social insurance physicians at the Institute, and performed face-to-face work disability assessment interviews. Staff/executive social insurance physicians and physicians who had been social insurance physicians for less than one year were not eligible for participation.

We decided that the Educational Department of the Institute would offer the training course. The infrastructure of the Educational Department was used to make the training course known, by approaching the managerial staff and presenting the training course to them to raise awareness and generate enthusiasm for the course among physicians. We also distributed flyers and newsletters with information about the training course, organised presentations for both social insurance physicians and their executives at four front offices of the Institute, provided information on the website of the research project, and sent e-mail invitations to physicians. Furthermore, physicians who were training to be registered social insurance physicians were approached by their educational institute with information, and informed that they could use the training course as an optional subject in their education. Finally, we sent e-mails to all the social insurance physicians who had previously participated in the research project (i.e. questionnaire studies or focus group study), or had previously shown interest in the study, informing them about the training course.

Two teachers, who were recruited from the Educational Department of the Institute, trained all groups of participants according to the detailed manual.

### Finding out whether the course increases knowledge and skills

A plan was made for future evaluation of the intervention in a two-armed RCT. This RCT has been registered in the Dutch Trial Register (NCT, number 2287). We will randomly assign participants to either an intervention group, or to a waiting list control group which will not participate in the training course until all measurements have been completed. Participants in the intervention group will complete a questionnaire at baseline and directly after the training course (1-2 weeks after baseline). Participants in the waiting list control group will complete the questionnaires at the same moments as the participants in the intervention group. The primary outcomes will be skills (measured with a casuistry example) and knowledge (measured with true-false questions) with regard to communication during work disability assessment interviews. Because it is known that improvements in knowledge are not necessarily accompanied by improvements in communication skills [27,28], a casuistry example will be included in the measurements. This will be a vignette of a sick-listed claimant applying for a disability benefit. It includes open-ended questions intended to see if physicians notice potential difficulties in the communication and if they know how to adjust the communication in order to prevent such difficulties. We consider this to be, given our practical constraints, the best approximation of real practice. A process evaluation will be carried out, to determine the most effective and valued aspects, to identify barriers and facilitators for implementation, and to further improve the training course. This will involve gathering data from the social insurance physicians (questionnaires) and the two teachers (informal interviews). If the results of the planned evaluation will be positive and show that the developed training is able to increase physicians' awareness of the communication as well as their communication skills, than additional evaluations will be planned. To add such an evaluation in practice is especially important, because the (changes in) opinions and experiences of claimants are the final test of the training's effectiveness.

## Discussion

### Main findings

Following the six steps of the Intervention Mapping protocol, we developed the 'Professional Claimant Communication' post-graduate training course, aimed at achieving professional physician-claimant communication during disability assessment interviews. The results of the first IM step indicated that the stakeholders would prefer a training course promoting a professional physician-claimant relationship, with clear, empathic, and non-biased communication. The second step resulted in the three main programme objectives, i.e. awareness of assumptions about claimants, communication attuned to claimants, and clarity concerning the findings of the assessment. For the change objectives, continuous support in realising and implementing the training course needs to be obtained from the Institute. Step 3 showed the importance of active teaching strategies, based on the Social Cognitive Theory and the model of learning styles. In the fourth IM step, it was established that the training course would be a two-day course that would follow the phases of an assessment interview (i.e. the introduction phase, the information-gathering phase, and the closing phase). Step 5 resulted in the use of the infrastructure of the Educational Department in making the training course known and its implementation. In the sixth and last step, a plan was formulated for an RCT with a waiting list control group to evaluate the training course.

There is increasing use of the Intervention Mapping protocol for the systematic development of training courses for medical professionals [16,17,29], but it is only recently that researchers have applied the protocol to develop interventions in the context of work disability assessments [16,29]. They found that the protocol, albeit extensive and time-consuming, is beneficial in this context. This is in agreement with our findings in the present study.

### Strengths and limitations of the study

The IM protocol is a substantive protocol, implying that a considerable amount of time is needed to develop an intervention. However, this time-investment seemed to be worthwhile, because the focus of the protocol on practicality and feasibility, as well as the participation of all directly concerned stakeholders in the development of the intervention, seem to have resulted in a training course with great potentials, but the disadvantage is that the training course is only relevant for physicians who perform work disability assessment interviews, and not for other physicians. On the other hand, Bos et al. [30] concluded that training courses need to be based on context-specific needs assessments, which is in agreement with the opinions of the experts who participated in the present study. A strength of this study is that the opinions and experiences of both the Institute and the claimants were used in the development of the training course, thus ensuring its practical relevance and feasibility.

Earlier studies have demonstrated that supervision provided by more experienced physicians is important to facilitate the transfer of the skills that have been learned into practice [31,32]. Although this was confirmed by the experts in our study, due to practical issues it could not be included in the training course. It would, however, be advisable to continue to search for possibilities in this respect. After all, the ultimate aim of such training courses is their generalisation to practice, so that claimants can benefit.

The systematic approach of the IM protocol ensures reproducibility, and therefore implies that the results would be comparable if other researchers performed the same study. However, the choices made in the initial IM steps are crucial, and therefore a difference in choices might result in a difference in emphasis in the development phase, and thus in a totally different training course. Although we made our choices (e.g. which stakeholders to include) very carefully, it is likely that there are also local differences among stakeholders, and that stakeholders of other origin (e.g. physicians or claimants in other countries) would have expressed other needs, resulting in different objectives. The degree of reproducibility is therefore dependent on the context and situation in which the development takes place, but because application of the IM protocol leads to a profound development of interventions, and ensures that no essential steps are left out, the chances of developing at a comparable intervention when including comparable stakeholders, are high.

All the measurements in our evaluation plan will be based on questionnaires. Various other methods of evaluation, such as systematic observations of video-recordings of real assessment interviews, or interviews with simulated claimants, might provide stronger evidence. However, due to practical issues, we had to choose a less time-consuming method of evaluation. We recommend that future studies should use more robust methods if possible. For example, an extensive RCT could be performed, in which the measurements include observations of actual physician-claimant interviews, instead of only "paper-and-pencil" measurements. Furthermore, the claimant's opinion about the communication skills of the physician could be taken into account.

### Implications

Work disability assessments are performed in many countries [1,3], and always have a big impact on disability claimants, even though the assessments are made in different legal contexts and different procedures may apply. However, when these assessments include face-to-face interviews, communication will obviously influence the process and content of the assessment. Despite common agreement on this matter, our study was the first - to our knowledge - in which an attempt was made to develop an evidence-based communication skills training course for physicians who regularly perform these assessments. This tailoring is especially important because of ethical issues related to work disability assessment interviews. Several of our findings with regard to the main objectives, the strategies, and the implementation plan, are relevant for use in other countries than the Netherlands. Preferably, however, the training course should be tailored to the specific practice of work disability assessment in those countries. However, because a large part of our training course focuses on knowledge, awareness, and the communication skills that are relevant for all physicians who perform work disability assessments, and are probably applicable to physicians worldwide, specific tailoring should not be difficult. Due to the fact that IM is not only a systematic, but also a circular approach, the results of our study can be used as a starting point in the tailoring process.

## Conclusions

The practical relevance and feasibility of our communication skills training course is promising. Its content is important for physicians in many different countries, and with IM the training course can be tailored to specific local practices. Moreover, the results of our study represent an important advancement in this field, because previously there was no such evidence-based training course available that was tailored to work disability assessments.

## Competing interests

The authors have no conflicts of interest that are relevant to the content of this article.

## Authors' contributions

All authors contributed to the design and performance of this study, and to the development of the communication skills training course. HJvR wrote the manuscript. AJMS, JRA, WELdB, and AJvdB commented on and revised the manuscript. All authors have read and approved the final version of the manuscript.

## Pre-publication history

The pre-publication history for this paper can be accessed here:

http://www.biomedcentral.com/1472-6920/11/28/prepub

## Supplementary Material

Additional file 1 **Summary of the training programme**. Click here for file
