# Supplementary material for: Systematic development of a communication skills training course for physicians performing work disability assessments: from evidence to practice
Source: BMC Med Educ. 2011 Jun 3;11:28. doi: 10.1186/1472-6920-11-28 (PMC3138427; doi:10.1186/1472-6920-11-28)
Supplement: Additional file 1 — Summary of the training programme. [file 1472-6920-11-28-S1.DOC]

# Appendix 1 – Summary of the training programme

**Manual Professional Claimant Communication**

- Day 1: Emphasise introduction of the interview and gathering information.
- Day 2: Emphasise gathering information and discussing the conclusion.
- Theme throughout the course: professional involvement.

**Day 1**

**9.30 hours – Getting acquainted**

*Teaching strategies: plenary*

- Teachers introduce themselves (formal/instrumental and empathic/affective).
- Teachers explain their roles: both teacher and role-play actor.
- All participants introduce themselves by answering three questions: (1) who are you (name, etc.)?, (2) do you have any experience with communication skills training courses and related education?, and (3) what do you hope to have learned by the end of this course? The aim is to provide structure and a safe environment.

**10.00 hours – Explanation of the programme, aims, and methods**

*Teaching strategies: plenary*

- Basis of the training course: every participant has his/her own personal expertise with regard to the performance of assessment interviews. Singularity of all participants is respected.
- The course topics are predetermined, but all participants will have the opportunity to practice with the situations that they find difficult.

**10.15 hours – Theoretical model (self-other model)**

*Teaching strategies: plenary, interactive*

- Short explanation of theory, applied to contact with claimants. The continuous interaction between the communication of the physician (self) and the claimant (other) is highlighted.
- Practice in application of the self-other model.

**10.45 hours – Preconditions for learning from the training course**

*Teaching strategies: plenary, interactive, summary on flip-over*

- Focus on safety in the group. What do participants need to participate comfortably?

**11.15 hours – Introduction of the assessment interview**

*Teaching strategies: role-play, video-recordings, brainstorming, group discussion, feedback, hand-outs*

- Theme: How do I introduce myself to the claimant, and how do I explain the aims of the assessment interview?
- Participants receive a vignette of a 55-year old construction worker with low back pain and mental complaints. He is surly and uncommunicative. They are not allowed to discuss this vignette with the other participants.
- Each individual participant gives a short introduction – no more than a few minutes – to the first assessment interview with the construction worker (enacted by one of the teachers), as the participant would do in every day practice. These role-plays are recorded.
- Before the group sees the video-recordings, all participants together make a list of criteria that are important in a first introduction.

**12.00 hours – Lunch break**

**13.00 hours –Introduction of the assessment interview (continued)**

- The Korthagen reflection circle is explained, as a method to give adequate feedback to fellow participants.
- The participants watch the video-recordings of the introductions and they are encouraged to give feedback to each other.
- The recordings are evaluated according to the list of criteria.

**13.45 hours – Gathering information: concise theory**

*Teaching strategies: plenary interactive teaching, group discussion, hand-outs*

- Both the content of the interview and the process (contact) are always important.
- Short explanation of the theory for each topic (see below). Participants are encouraged to ask questions.
- Short practice of role-play for each topic (one of the teachers plays the role of the claimant).

Topics:

- Types of questions (open-ended, closed, normative, ‘why’ questions).
- Listening, summarising, asking follow-up questions.
- Using silences as a conversation technique.
- Shifting between the content and the process (contact) of communication.

**14.30 hours – Practising assessment interviews**

*Teaching strategies: plenary role-play, feedback from participants and teachers*

- All participants get a chance to practice.
- Main focus on gathering information with an uncommunicative and not forthcoming claimant, with emphasis on the four topics mentioned above.
- Participants are allowed to practise with the types of claimants that they have trouble with and/or situations in the assessment interview that they find difficult, if these are relevant for the topic.
- The following practise cycle is repeated several times:
  - One of the teachers or participants presents a (difficult) situation in the information-gathering phase of an assessment interview.
  - A short role-play takes place, in which one of the teachers plays the role of the claimant. The other participants observe, focusing on the topics mentioned above, and the feedback that should be given on the performance concerning those topics.
  - The participant in the role-play gets the opportunity to reflect on his/her performance in the role-play situation.
  - The other participants and the teacher give feedback on what went well and what could be improved.
  - Another participant, or several other participants, can practise the same situation and receive feedback.

**16.15 hours – Review of day 1**

*Teaching strategies: plenary*

- Feedback to teachers. Things that need to be shared.

**16.30 hours – End of day 1**

**Day 2**

**9.30 hours – Review of day 1 and preview of day 2**

*Teaching strategies: plenary*

**10.00 hours – Non-verbal behaviour**

*Teaching strategies: plenary interactive teaching, group discussion, hand-outs*

- Explanation and discussion of how and what non-verbal behaviour contributes to the assessment interview. What a physician can do with the non-verbal behaviour of a claimant. How to interpret non-verbal behaviour. How to give proper feedback.

*Teaching strategies: plenary role-play, feedback from participants and teachers*

- Practising assessment interviews with a focus on non-verbal behaviour of both physician and claimant:
  - One of the teachers or participants presents a (difficult) situation in the information-gathering phase of an assessment interview.
  - There is a short role-play, in which one of the teachers plays the role of the claimant. The other participants observe, focusing on the topics mentioned above and what feedback should be given on the performance concerning those topics.
  - The role-playing participant has the opportunity to reflect on his/her performance in the role-play situation.
  - The other participants and the teacher give feedback on what went well and what could be improved.
  - Another participant, or several other participants, can practise the same situation and receive feedback.

**10.30 hours – Giving and receiving feedback in an assessment interview**

*Teaching strategies: plenary interactive teaching, group discussion, hand-outs, plenary role-play, feedback from participants and teachers*

- Discussing theory about feedback.
- Practicing assessment interviews with a focus on giving and receiving feedback. The same procedure is applied as described for ‘non-verbal behaviour’.
- Both positive and negative feedback: compliments and criticism.

**11.00 hours – Discussing the conclusions of the assessment interview**

*Teaching strategies: plenary interactive teaching, group discussion, hand-outs, plenary role-play, feedback from participants and teachers*

- Discussing theory about sharing conclusions with claimants, based on methods for breaking bad news. The focus is on situations in which the claimant disagrees with the physician about the conclusion.
- Practising assessment interviews, with a focus on discussing the conclusions of the assessment and breaking bad news. The same procedure is applied as described for ‘non-verbal behaviour’.

**12.00 hours – Lunch break**

**13.00 hours – Discussing the conclusions of the assessment interview (continued)**

*Teaching strategies: plenary role-play, feedback from participants and teachers*

- Practising the closing of assessment interviews and discussing the conclusions of the assessment with claimants, thereby paying attention to breaking bad news, non-verbal behaviour, and giving feedback. The same procedure is applied as described for ‘non-verbal behaviour’.

**15.00 hours – Personal Action Plan**

*Teaching strategies: plenary quiescence, group discussion*

- Each participant thinks in silence about what he or she will do differently in the next assessment interview, after this two-day training course. These intentions should be well-defined and concrete.
- Each participant explains his or her intentions to the other participants.

**16.00 hours – Review of days 1 and 2**

Oral and written evaluation.

**16.30 hours – End of the training course**
